# Acidic extracellular pH induces autophagy to promote anoikis resistance of hepatocellular carcinoma cells via downregulation of miR-3663-3p

**DOI:** 10.7150/jca.51849

**Published:** 2021-04-19

**Authors:** Siying Wang, Yuanyuan Lv, Yangyang Zhou, Jing Ling, Hui Wang, Dishui Gu, Cun Wang, Wenxin Qin, Xingling Zheng, Haojie Jin

**Affiliations:** 1 State Key Laboratory of Oncogenes and Related Genes, Shanghai Cancer Institute, Renji Hospital, Shanghai Jiao Tong University School of Medicine, No. 25/Ln 2200 Xie-Tu Road, Shanghai 200032, China.; 2 Department of Pathophysiology, School of Basic Medical Sciences, Guangdong Medical University, Dongguan, Guangdong, 523808, China.

**Keywords:** hepatocellular carcinoma, acidic tumor microenvironment, anoikis resistance, autophagy, miR-3663-3p

## Abstract

Metastasis is the major reason for poor prognosis and high fatality in hepatocellular carcinoma (HCC). Due to the “Warburg effect”, an acidic tumor microenvironment (TME) exists in solid tumors and plays a critical role in cancer metastasis. Thus, clarifying the mechanism underlying the acidic TME in tumor metastasis could facilitate the development of new therapeutic strategies for HCC. Anoikis resistance is one of the most important events in the early stage of cancer metastasis. Here, we report that acidic extracellular pH (pH_e_) promotes autophagy of HCC cells via the AMPK/mTOR pathway. We found that autophagy induced by acidity enhances anoikis resistance of HCC cells, which could be reversed by autophagy inhibitors. Furthermore, miR-3663-3p was downregulated by acidity, and overexpression of miR-3663-3p abolished acidic pH_e_-induced autophagy and anoikis resistance. In summary, acidic pH_e_ enhances anoikis resistance of HCC cells by inducing autophagy, which is regulated by miR-3663-3p. Our findings provide new insight into how the acidic TME is involved in HCC progression.

## Introduction

Hepatocellular carcinoma (HCC) is the most common primary liver cancer, with second leading cause of cancer-related deaths worldwide 1. The prognosis of HCC is dismal with a 3-year survival rate of 12.7% and a median survival of 9 months 2. Metastasis is the major cause of poor prognosis and high fatality in HCC patients. Therefore, it is pivotal to elucidate the molecular mechanisms underlying metastasis in HCC.

Tumor microenvironment (TME) is closely associated with tumor metastasis. Characteristics of TME include acidity, hypoxia, reduced glucose concentrations, secretome changes, and recruitment of stromal and immune cells 3-7. Among these features, the acidic TME is considered as a driver of cancer 4. Several evidences indicates that acidosis affects epithelial-to-mesenchymal transition (EMT) and extracellular matrix (ECM) remodeling in different tumors. For example, melanoma cells subjected to extracellular acidosis exhibit increased expression of EMT markers and augmented invasiveness* in vitro* and *in vivo* 8. In pancreatic cancer cells, acid-induced stimulation of acid-sensing ion channels could positively regulate acidity-induced EMT 9. A moderately acidic TME can promote the migration and invasion of HCC cells 10. Our previous work also indicated that an acidic TME can activate hepatic stellate cells and promote the metastasis of HCC 11.

Anoikis is defined as the induction of apoptosis due to detachment from the ECM 12, and cancer cells must develop resistance to anoikis for tumor metastasis 13. Anoikis resistance is associated with authophagy, which recycles macromolecules to give cells an advantage for survival under stressful conditions, such as nutrient starvation, oxidative stress, hypoxia, and metabolic stress 14. Loss of attachment-induced autophagy can increase anoikis resistance in lung cancer cells 15. Knockdown of BECN1, one major mediator of autophagy, has been demonstrated to inhibit anoikis resistance in models of peritoneal carcinomatosis and sarcomatosis 16. Autophagy inhibition decreases pulmonary metastasis of HCC in mice by attenuating anoikis resistance and lung colonization 17. These evidence indicates that autophagy plays a role in the anoikis resistance of tumors. However, the effects of an acidic microenvironment on autophagy and anoikis resistance and the underlying mechanisms have not been fully elucidated in HCC yet.

In the present study, we mimicked the acidic TME by using a low pH medium, and found that acidic extracellular pH (pH_e_) could induce autophagy and contribute to anoikis resistance of HCC cells. Furthermore, we identified downregulation of miR-3663-3p enhanced the anoikis resistance of HCC cells in the condition of acidic pH_e_. Overall, our study demonstrates that acidic TME confers HCC cells anoikis resistance via downregulation of miR-3663-3p, and finally drives HCC metastasis.

## Material and methods

### Cell culture

Human HCC cell line MHCC97H was provided by the Liver Cancer Institute of Zhongshan Hospital of Fudan University (Shanghai, China). Huh7 cells were purchased from Riken Cell Bank (Tsukuba, Japan) and SMMC7721 cells from Cell Bank of Shanghai Institute of Biochemistry and Cell Biology, Chinese Academy of Sciences (Shanghai, China). All of the cell lines were cultured in Dulbecco's modified Eagle medium (DMEM; Gibco, CA, USA) containing 10% fetal bovine serum (Gibco, CA, USA) in a 5% CO_2_ atmosphere at 37 °C. The medium was further supplemented with 25 mM HEPES (Sigma-Aldrich, MO, USA) and 1.5 g/L NaHCO_3_ buffer, and adjusted to pH 7.4 in the 5% CO_2_ atmosphere at 37 °C. The acidic medium (pH 6.6) was prepared just like the medium of pH 7.4, except it was supplemented with extra 25 mM MES (Sigma-Aldrich, MO, USA).

### Transfection of oligonucleotides

miRNA mimics and inhibitors were synthesized by Ambion (Austin, USA). Cells were transfected with the oligonucleotides using Lipofectamine® RNAiMAX reagent (Invitrogen) at a final concentration of 50 nM and used for further experiments after 48 hours.

### Western blot

Tissue and cell samples were homogenized in a RIPA buffer (Qiagen, China) with a cocktail of proteinase inhibitors (Roche Applied Science, Switzerland) and phosphatase inhibitors (Roche Applied Science, Switzerland). Protein concentrations were determined using the Bicinchoninic Acid (BCA) Kit (Pierce). Proteins were separated by SDS-PAGE and transferred to nitrocellulose membranes (Bio-Rad, Hercules, CA), which were incubated with primary antibodies for LC3-I/II (CST, #4108, 1:1000), p62 (CST, #88588, 1:1000), p-AMPKα1/2 (R&D, AF2509, 1:2000), t-AMPKα (CST, #5831,1:1000), p-mTOR (CST, #5536, 1:1000), t-mTOR (CST, #2972, 1:1000), cleaved caspase-3 (CST, #9664, 1:1000), PARP (CST, #9532, 1:1000), Bcl-xl (CST, #2764, 1:1000), Mcl-1 (CST, #94296, 1:1000), or β-Actin (Sigma, A2066, 1:2000) overnight at 4 °C and probed with secondary antibodies at room temperature for 1~2 h. After washing the membranes, an enhanced chemiluminescence detection (ECL) reagent was used to expose the protein bands in a digital chemiluminescence imaging system (Bio-Rad).

### Real-time polymerase chain reaction assay

Total RNA was extracted from cells and tissues using TRIzol reagent (Invitrogen, Carlsbad, CA) and reverse transcription performed using the PrimeScript™ RT Reagent Kit (Takara, Dalian, China) according to the manufacturer's instructions. Quantitative real-time polymerase chain reaction (PCR) was subsequently performed with the SYBR Premix Ex Tag (Takara, Dalian, China) according to the manufacturer's instructions. Data were normalized to β-actin. The primers are given in Supplementary Table 1.

### Immunofluorescence

Tumor cells were fixed with 4% paraformaldehyde, permeabilized with 0.1% Triton X-100 for 10 min, and then blocked with 5% bovine serum albumin (Amresco, OH, USA). The cells were incubated with the primary LC3-I/II antibody (CST, #4108, 1:100) overnight at 4 °C. After washing with PBS, the cells were incubated with Alexa-flour 594 secondary antibody (Invitrogen) at room temperature for 1 h. Nuclei were stained with DAPI (Sigma) for 5 min. Following three rinses with PBS, the cells were imaged by confocal laser scanning microscopy (FluoView FV1000, Olympus).

### Transmission electron microscopy

Tumor cells cultured in medium of pH 6.6 or pH 7.4 for 48 hours were collected and fixed with 2.5% glutaraldehyde at 4 °C. Glutaraldehyde fixative was then removed and tumor cells were fixed with 1% osmium tetroxide (OsO4) at 4 °C for 2 hours. After ethanol gradient dehydration, slices 60-nm-thick were cut. Uranyl acetate-lead citrate was applied to re-dye the slices. The cells were observed with a transmission electron microscope (Philips, CM100). Autophagic cells were characterized by many autophagosomes enwrapping organelles.

### Quantitative real-time PCR of miRNAs

Tumor cells were cultured in medium of pH 6.6 or pH 7.4 for 48 h and then total RNA was extracted using the miRNeasy Mini Kit (Qiagen, Hilden, Germany) according to the manufacturer's protocol. Complementary DNA was synthesized using the PrimeScript RT reagent Kit (TaKaRa, Tokyo, Japan). Mature miRNAs were quantified with specific primers and probes using TaqMan MicroRNA Assays (Applied Biosystems, Foster City, USA). U6B small nuclear RNA (snRNA) was used as a loading control.

### Caspase-3/7 activity assay

Cells were treated with miR-3663-3p mimic or control (50 nM) and cultured in pH 6.6 or pH 7.4 medium. Then cells were plated on poly-HEMA-coated plates to prohibit attachment. After 48 hours in suspension, caspase-3/7 activity was measured by the Caspase-Glo 3/7 assay (Promega, Madison, WI, USA, G8091) according to the manufacturer's protocol.

### Apoptosis assays

Cells were treated with 3-MA (5 mM) or CQ (50 nM) and then cultured in medium of pH 6.6 or pH 7.4. Then cells were plated onto poly-HEMA-coated plates. After 48 hours in suspension, apoptotic cells were quantified (percentage) using an Annexin V-fluorescein isothiocyanate (FITC)/propidium iodide (PI) apoptosis detection kit (BD). The cells were harvested and counted using the TC10 Cell Counter (Bio-Rad, USA).

### Microarray processing

Total RNA was extracted from specimens using TRIzol reagent (Invitrogen, MA, USA) in accordance with the manufacturer's protocol. The quantity and quality of RNA were evaluated using an Agilent 2100 Bioanalyzer (Agilent Technologies, CA, USA). Microarray hybridization, scanning, and analysis were performed by Shanghai Biotechnology Corporation using Agilent Human miRNA (8*60K) arrays (Shanghai Biotechnology Corporation). Briefly, 2 µg of sample RNA was directly labeled with biotinylated signaling molecule using FlashTag Biotin HSR RNA Labeling kits (Affymetrix, USA). The chips were hybridized and washed in accordance with the manufacturer's protocol. Scanning was performed using GeneChip Scanner 3000. GeneChip Operating Software was used to analyze the data. Average log2 ratios were calculated from the normalized data for each miRNA.

### Statistical analysis

Differences among variables were assessed by χ^2^ analysis or a two-tailed Student *t* test. Data were presented as mean ± SEM. Differences were considered to be significant if *p*<0.05.

## Results

### Acidic pH_e_ induced autophagy of HCC cells

**Figure 1:**
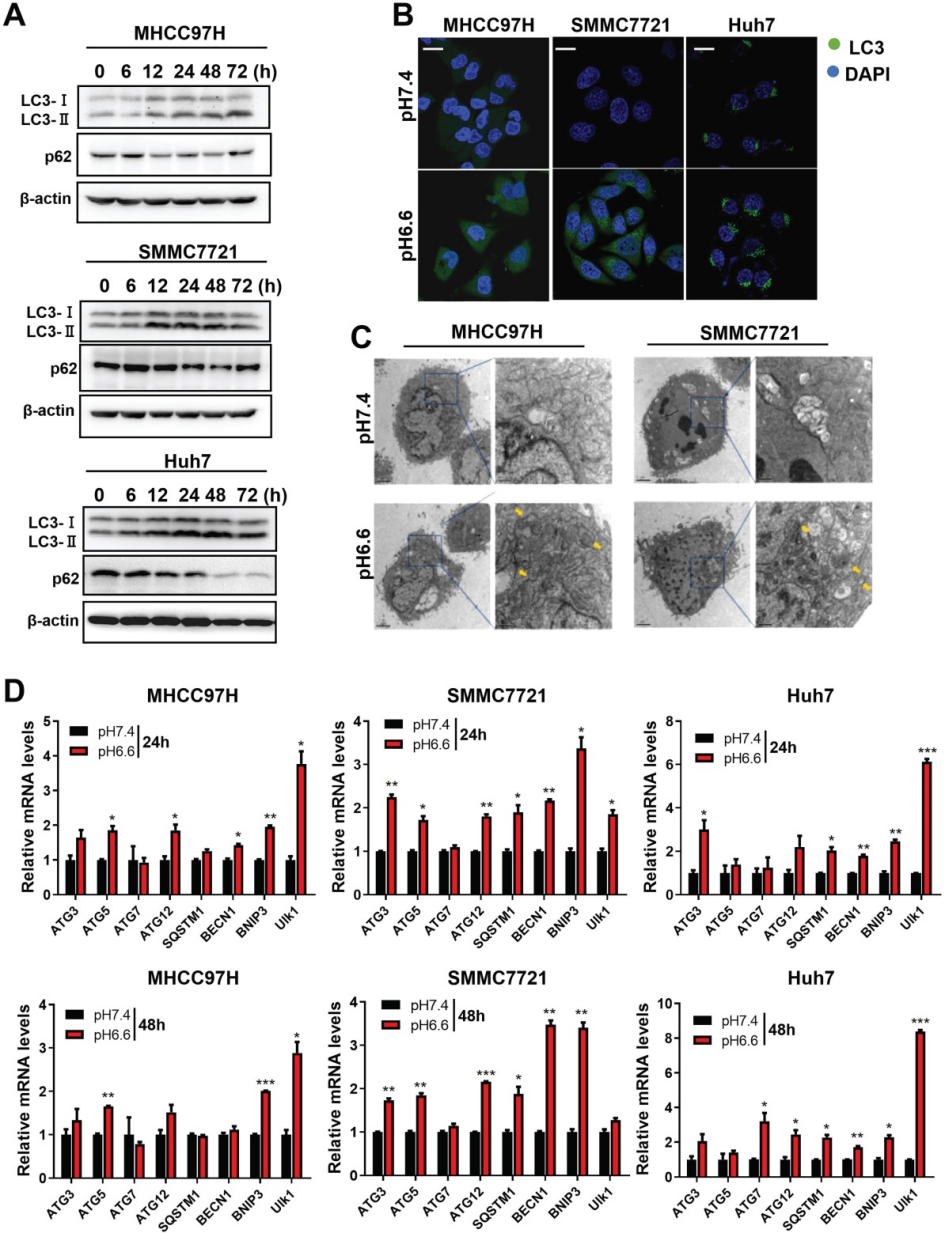
Acidic pHe induced autophagy of attached HCC cells. (A) Western blots of protein levels of autophagy markers LC3-Ι, LC3-II, and p62 at different time points in HCC cells treated with medium of pH 6.6. (B) HCC cells were stained with fluorescent antibodies against LC3 (green) or with DAPI (blue). Representative confocal immunofluorescence images are shown. Scale bars: 20 µm. (C) Representative images of transmission electron microscopy of autophagic ultrastructural features in HCC cells. Arrows indicate autolysosomes and autophagosomes. Scale bars: 2 µm (left) or 0.5 µm (right). (D) Quantification of the mRNA levels of autophagy-related proteins evaluated by real-time qRT-PCR of HCC cells treated with medium of pH 7.4 or pH 6.6 for 24 hours (left) or 48 hours (right).

We mimicked an acidic TME by culturing HCC cells in medium of pH 6.6 and investigated autophagy status. We found that protein levels of LC3-I and LC3-II increased, whereas protein levels of p62 decreased in a time-dependent pattern over 72 hours when normal culture medium was replaced by acidic medium (Fig. 1A). Confocal immunofluorescence further confirmed increased protein levels of LC3 after culturing with acidic medium (Fig. 1B). Transmission electron microscopy showed increasing autolysosomes or autophagosomes after acidic medium treatment (Fig. 1C). In addition, autophagy-associated genes were upregulated at the mRNA level in HCC cells when cultured in acidic medium for 24 or 48 hours (Fig. 1D). These data indicate acidic pH_e_ could promote autophagy of HCC cells.

### Acidic pH_e_ induced autophagy of HCC cells through AMPK and mTOR signaling pathways

**Figure 2:**
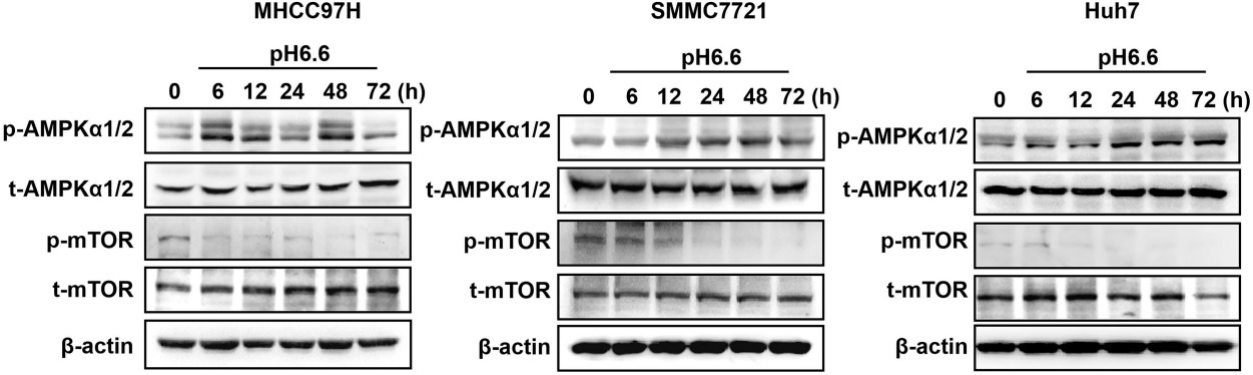
Acidic pH_e_ activated AMPK signaling and inhibited mTOR signaling in attached HCC cells. Western blots of the levels of p-AMPKα1/2, t-AMPKα1/2, p-mTOR, and t-mTOR at different time points are shown.

Mammalian target of rapamycin (mTOR) is a major signaling pathway controlling autophagy and regulated by the energy state of the cells. When energy becomes limiting, the inhibitory effect of mTOR decreases and autophagy is induced. Adenosine monophosphate-activated protein kinase (AMPK) is a major regulator of autophagy in both yeast 18 and mammalian cells 19, 20. When the level of ATP falls, the increase in AMP stimulates AMPK and results in the inhibition of mTOR. Considering the significance of mTOR signaling and AMPK signaling in autophagy regulation, we analyzed key components of the AMPK/mTOR signaling pathway in HCC cells cultured in acidic medium. The results showed that acidic medium treatment strongly activated the levels of phosphorylated AMPKα1/2 and subsequently decreased the levels of phosphorylated mTOR decreased (Fig. 2), which probably leading to autophagy induction in HCC cells.

### Acidic pH_e_-induced autophagy promoted anoikis resistance of HCC cells

**Figure 3:**
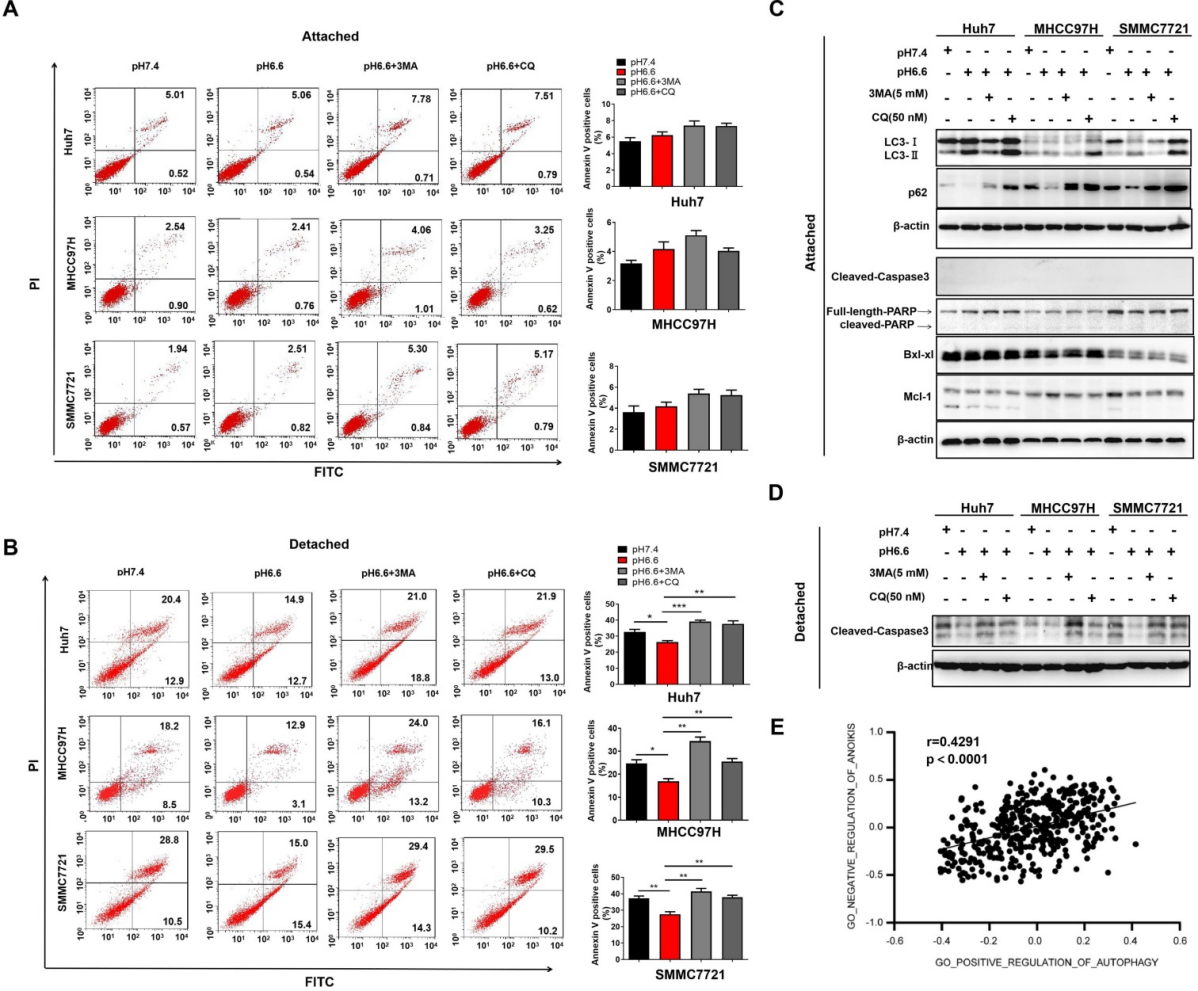
Acidic pH_e_-induced autophagy promoted anoikis resistance of HCC cells. (A) Attached HCC cells were treated with medium of pH 7.4 or pH 6.6. Cells in medium of pH 6.6 were treated with 3-MA (5 mM) or CQ (50 nM). Survival of the attached cells was analyzed by flow cytometry. (B) Detached HCC cells were treated with medium of pH 7.4 or pH 6.6. Cells in medium of pH 6.6 were treated with 3-MA (5 mM) or CQ (50 nM). Survival of the detached cells was analyzed by flow cytometry. (C) The levels of autophagy-related markers and apoptosis-related markers in attached cells were analyzed by Western blot. (D) The levels of cleaved-caspase 3 in detached cells were analyzed by Western blot. (E). Correlation analysis of the gene signature of GO_NEGATIVE_REGULATION_OF_ANOIKIS and gene signature of GO_POSITIVE_REGULATION_OF_AUTOPHAGY in HCC TCGA datasets. Data are shown as the mean±SEM of three independent experiments. *p<0.05, **p<0.01, ***p<0.001.

Autophagy contributes to the survival of tumor cells, which are lack of appropriate matrix contact, either during early carcinoma formation or in the later stages of dissemination and metastasis 21, 22. To induce anoikis, we cultured HCC cells in attachment-free conditions on poly-HEMA coated plates. Cells cultured in attachment conditions on regular plates served as a control. An acidic environment had no obvious effect on apoptosis in attached cells, with or without the treatment of autophagy inhibitors, 3-methyladenine (3-MA) or chloroquine (CQ) (Fig. 3A). In contrast, acidic medium significantly facilitated anoikis resistance of detached cells, and blocking autophagy could reverse the effect (Fig. 3B). Molecular markers associated with autophagy and apoptosis in the attached cells and detached cells further confirmed the results (Fig. 3C, D). Moreover, correlation analysis in TCGA cohort also showed that autophagy was significantly associated with negative regulation of anoikis (Fig. 3E). These results indicate that acidic pH_e_ could enhance anoikis resistance by inducing autophagy.

### Acidic pH_e_ induces autophagy via downregulation of miR-3663-3p in HCC cells

**Figure 4:**
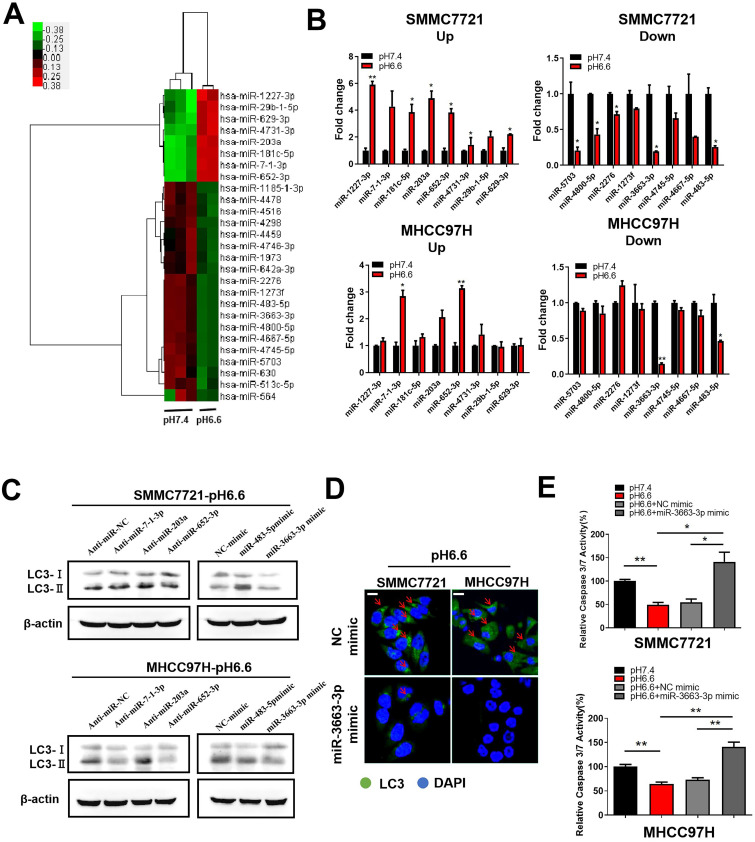
Acidic pHe-induced autophagy protected HCC cells from anoikis via downregulation of miR-3663-3p. (A) Heat map of miRNA expression profiles of attached SMMC7721 in medium of pH 7.4 and pH 6.6 (fold change > 2.0 or fold change < -2.0, q value < 0.05). (B) Fold changes in the top 8 upregulated (left) and top 8 downregulated (right) miRNAs in attached HCC cells verified by real-time qRT-PCR. (C) HCC cells in medium of pH 6.6 were transfected with anti-miRNAs or miRNA mimics and the protein levels of autophagy-related markers LC3-Ι and LC3-II detected by Western blot. (D) HCC cells in medium of pH 6.6 were transfected with miR-3663-3p or NC mimics and stained with fluorescent antibodies against LC3 (green) or with DAPI (blue). Representative confocal immunofluorescence images are shown. Red arrows indicate the cells with obvious autophagosomes. Scale bars: 20 µm. (E) HCC cells in medium of pH 6.6 were transfected with miR-3663-3p or NC mimics and the relative caspase 3/7 activity analyzed in detached cells. Data are shown as the mean±SEM of three independent experiments. *p<0.05, **p<0.01.

More and more evidence showed that miRNAs, sucha as miR-138-5p 23, miR-30b 24, and miR-224-3p 25, can take part in the regulation of autophagy in cancer cells by targeting autophagy-associated genes. We also previously discussed the potential characteristics of miRNAs in hypoxia and acidic TME 26. To explore the molecular mechanism by which acidic TME regulates autophagy in HCC cells, we compared miRNA expression profiles in HCC cells cultured in medium of pH 6.6, which mimics an acidic TME, to those in normal medium of pH 7.4. The miRNA expression profile analysis revealed 27 dysregulated miRNAs (fold change > 2.0 or fold change <-2.0, q value <0.05), 8 of which were upregulated and 19 downregulated (Fig. 4A). We verified the expression of top 8 upregulated miRNAs and top 8 downregulated miRNAs by qRT-PCR. MiR-7-1-3p, miR-203a, and miR-652-3p were prominently upregulated, whereas miR-3663-3p and miR-483-5p were significantly downregulated in both SMMC 7721 and MHCC 97H cells (Fig. 4B). Subsequently, we assessed the ability of these miRNAs to regulate autophagy and found that miR-3663-3p might be involved in acidic TME-induced autophagy. The results showed that overexpression of miR-3663-3p mimics decreased autophagy, as indicated by levels of autophagy-related markers LC3-I and LC3-II (Fig. 4C, D). Moreover, manipulation of miR-3663-3p was able to recover relative caspase 3/7 activity of HCC cells, which was downregulated by acidity (Fig. 4E), indicating that miR-3663-3p negatively regulated anoikis resistance. Taken together, we speculated that acidity may induce autophagy by downregulating miR-3663-3p, and finally inhibit anoikis.

## Discussion

An acidic TME favors the EMT and dissemination of cancer cells, which are prerequisites for invasive behavior 27. We previously found that an acidic TME could activate hepatic stellate cells and promote the metastasis of HCC 11. In this study, we demonstrated that acidic pH_e_ induced autophagy of HCC cells through the AMPK and mTOR signaling pathways, and autophagy induced by acidic pH_e_ could inhibit anoikis in detached HCC cells rather than apoptosis in attached cells. MiR-3663-3p participated in the effects of acidic pH_e_ on autophagy and anoikis resistance of HCC cells.

HCC shows a characteristic TME due to its distinctive metabolic reprogramming. As the major metabolic organ in the body, liver plays an important role in glucose homeostasis by regulating synthesis and decomposition of glycogen. Compared to normal liver tissue, HCC shows elevated glycolysis, resulting in increased production of lactate. For example, upregulation of lactate dehydrogenase A (LDHA) and pyruvate dehydrogenase kinase (PDK) synergistically promotes the production of lactate in HCC 28, 29. Meanwhile, suppression of gluconeogenesis in HCC may contribute to lactate accumulation, which uses lactate as one of the substrates to consume harmful byproducts of glycolysis as actually a reverse pathway of glycolysis. The decrease of downstream gluconeogenesis enzymes, such as phosphoenolpyruvate carboxykinase1 (PCK1) and fructose-1,6-bisphosphatase 1 (FBP1) expression, in HCC lead to the suppression of gluconeogenesis and elevation of glycolysis 30, 31. One of the outstanding features of HCC is its strong association with liver fibrosis, with 80-90% of HCC developing in fibrotic or cirrhotic livers 32, and the surrounding fibrotic tissue contribute to highly hypoxic environment in HCC 33. Hypoxia-induced factor 1α (HIF-1α) is the primary factor in liver cancer hypoxia. As previously described, accumulation of HIF-1α may influence TME of HCC via the following four aspects. Firstly, HIF-1α can increase the uptake of glucose by upregulating the expression of glucose transporters (GLUT) such as GLUT1 34. Secondly, HIF-1α promotes the expression of glycolytic enzymes and accelerates the conversion of glucose to pyruvate 35. Thirdly, HIF-1α can phosphorylate pyruvate dehydrogenase (PDH) by inducing the expression of pyruvate dehydrogenase kinase (PDK) and inactivate the PDH to prevent the conversion of pyruvate to acetyl CoA 36. Last but not least, HIF-1α upregulates the expression of lactate dehydrogenase A (LDHA) to stimulate the production of lactic acid 37. Taken together, hypoxia interacts with glucometabolic reprogramming to contribute to a unique acidic TME in HCC.

Different animal models have shown that tumor pH_e_ ranges between 5.9 and 7.2 38-40. To explore the behavior of HCC cells in acidic TME, we cultured HCC cells in medium of pH 6.6 41, 42. We focused on the effect of acidic pH_e_ on autophagy in HCC cells by detecting molecular and morphological changes associated with autophagy. Acidic pH promoted autophagy of HCC cells *in vitro*. Similar findings were observed in other cancer types *in vivo*. Wojtkowiak JW et al. 43 confirmed that extracelluar pH can induce autophagy in MDA-MB-231 tumors and HS766T pancreatic cancer cells *in vivo* by positive pixel analysis of LC3 staining intensity in tumors. Pellegrini P et al. 44 also observed that increased autophagic compartment or the presence of a higher number of autophagic vesicles in the hypoxic/acidic areas in HCT116 tumor sections *in vivo*. However, how acidic pH regulates autophagy in HCC cells is unclear. mTOR plays a negative role in autophagy by regulating autophagy-related proteins and lysosome biosynthesis, and is subject to a variety of different upstream signaling pathways that can inhibit or promote autophagy levels by regulating mTOR. Under energy shortage conditions, mTOR is an important downstream target of AMPK 45, which can activate autophagy by indirectly activating ULK1 via the AMPK/mTOR pathway 46, 47. Autophagy mediated by the AMPK/mTOR pathway plays a key role in the treatment of hepatic ischemia-reperfusion injury in the numerous upstream pathways of autophagy 48. Ischemia and low perfusion contribute to the formation of acidic TME. In this study, we observed that acidic pH_e_ induced autophagy through the AMPK/mTOR pathway in HCC cells. Other important regulatory pathways upstream of autophagy, such as PI3K/AKT/mTOR, may play pivotal roles in the development of cancer. The roles of these pathways in acidic pH_e_-induced autophagy in HCC require further investigation.

Loss of adhesion promotes autophagy and cell survival. For example, protective autophagy in GSCs can promote resistance to anoikis 49. This suggests that autophagy may function as an anoikis-resistance mechanism in detached cells. Autophagy is involved in HCC metastasis by facilitating anoikis resistance and lung colonization by HCC cells 17. AEG-1 is a key contributor to anoikis resistance and metastasis by inducing autophagy in HCC 22. These reports provide evidence for autophagy triggered by different mechanisms to enhance anoikis resistance of tumor cells, but whether it happens in an acidic TME is unknown. Taken together, our data for the first time suggest that acidic pH_e_ can enhance anoikis resistance by inducing autophagy in HCC.

Mechanisms by which an acidic TME enhances anoikis resistance by inducing autophagy in HCC cells remain unknown. In metastatic HCC, miR-30a mediates Beclin 1 and Atg5-dependent autophagy, which confers anoikis resistance of HCC cells 50, suggesting important roles of miRNAs in the regulation of autophagy and anoikis resistance in cancer. We found that miR-3663-3p regulated autophagy in acidic pH_e_, which could affect anoikis in detached cells. Downregulation of miR-3663-3p has been reported in aneurysmal walls 51. Recently, a study described that LncRNA RPL34-AS1 can competitively bind miR-3663-3p and inhibit cell proliferation and invasion to promote apoptosis by regulating miR-3663-3p/RGS4 in papillary thyroid cancer cells 52. Our data suggest that miR-3663-3p participates in the regulation of acidic pH_e_-induced autophagy and anoikis. The detailed molecular mechanisms by which miR-3663-3p regulates autophagy and anoikis resistance in an acidic environment need to be further explored in HCC.

Autophagy inhibition or modifying one or more of the TME components has been considered as a new strategy for the treatment of tumors. Researchers tried to utilize the autophagy inhibitors, such as chloroquine (CQ), to block autophagic flux for tumor suppression 53. However, an acidic pH_e_ microenvironment was found to significantly inhibit the therapeutic efficacy of CQ 54, which supports that acidic pH_e_ could enhance the autophagy process. Our current study further confirmed that acidic pH_e_ could induce autophagy and promote anoikis resistance, resulting in enhanced metastasis. Thus, jointly targeting acidic TME could improve the therapeutic efficacy of autophagy inhibitors. Systemic treatment with sodium bicarbonate was proved to efficiently increase intratumoral pH and block tumor autophagy *in vivo* 43. Also, MnO_2_ could efficiently react to the excessive H^+^ in the TME and modulate acidic tumor microenvironments 55, 56. Based on the above findings, the conjunctive use of modulating acidic TME and autophagy inhibitors might be more effective for cancer therapy.

In conclusion, we found that acidic pH_e_ can enhance anoikis resistance by inducing autophagy through miR-3663-3p. This study is the first to reveal the roles of autophagy and anoikis resistance in an acidic microenvironment in HCC cells. Our findings indicate a potential approach for HCC treatment by targeting acidic TME and autophagy.

## Supplementary Material

Supplementary table S1.

## Abbreviations

HCC: hepatocellular carcinoma
TME: tumor microenvironment
EMT: epithelial-to-mesenchymal transition
ECM: extracellular matrix
pH_e_: extracellular pH
miRNA: microRNA
mTOR: mammalian target of rapamycin
AMPK: adenosine monophosphate-activated protein kinase
3-MA: 3-methyladenine
CQ: chloroquine
