# Supplementary material for: Acidic extracellular pH induces autophagy to promote anoikis resistance of hepatocellular carcinoma cells via downregulation of miR-3663-3p
Source: J Cancer. 2021 Apr 19;12(12):3418–26. doi: 10.7150/jca.51849 (PMC8120191; doi:10.7150/jca.51849)
Supplement: Supplementary file 1 — Supplementary table S1. [file jcav12p3418s1.pdf]

Supplementary Table 1. Primers used in this study

Primers for quantitative real-time PCR	Primer sequences (5'-3')
ATG3-forward	GACCCCGGTCCTCAAGGAA
ATG3-reverse	TGTAGCCCATTGCCATGTTGG
ATG5-forward	AGAAGCTGTTTCGTCCTGTGG
ATG5-reverse	AGGTGTTTCCAACATTGGCTC
ATG7-forward	CAGTTTGCCCCTTTTAGTAGTGC
ATG7- reverse	CCAGCCGATACTCGTTCAGC
ATG12-forward	CTGCTGGCGACACCAAGAAA
ATG12-reverse	CGTGTTCGCTCTACTGCCC
SQSTM1-forward	GACTACGACTTGTGTAGCGTC
SQSTM1-reverse	AGTGTCCGTGTTTCACCTTCC
BECN1-forward	GGTGTCTCTCGCAGATTCATC
BECN1-reverse	TCAGTCTTCGGCTGAGGTTCT
BNIP3-forward	CAGGGCTCCTGGGTAGAACT
BNIP3-reverse	CTACTCCGTCCAGACTCATGC
Ulk1-forward	GGCAAGTTCGAGTTCTCCCG
Ulk1-reverse	CGACCTCCAAATCGTGCTTCT
β-Actin-forward	TTGTTACAGGAAGTCCCTTGCC
β-Actin-reverse	ATGCTATCACCTCCCCTGTGTG
